# Green tea increases anti-inflammatory tristetraprolin and decreases pro-inflammatory tumor necrosis factor mRNA levels in rats

**DOI:** 10.1186/1476-9255-4-1

**Published:** 2007-01-05

**Authors:** Heping Cao, Meghan A Kelly, Frank Kari, Harry D Dawson, Joseph F Urban, Sara Coves, Anne M Roussel, Richard A Anderson

**Affiliations:** 1 Nutrient Requirements and Functions Laboratory, Beltsville Human Nutrition Research Center, Agricultural Research Service, US Department of Agriculture, Building 307C, BARC-East, 10300 Baltimore Avenue, Beltsville, MD 20705, USA; 2 Office of Clinical Research, National Institute of Environmental Health Sciences, National Institutes of Health, Department of Health and Human Services, Research Triangle Park, NC 27709, USA; 3 Unilever France, F92842 Rueil Malmaison, France; 4 Laboratoire de NVMC (Nutrition, Vieillissement et Maladies Cardiovasculaires), Faculte de Pharmacie, Joseph Fourier University, Domaine de la Merci, 38700 La Tronche, France

## Abstract

**Background:**

Tristetraprolin (TTP/ZFP36) family proteins have anti-inflammatory activity by binding to and destabilizing pro-inflammatory mRNAs such as Tnf mRNA, and represent a potential therapeutic target for inflammation-related diseases. Tea has anti-inflammatory properties but the molecular mechanisms have not been completely elucidated. We hypothesized that TTP and/or its homologues might contribute to the beneficial effects of tea as an anti-inflammatory product.

**Methods:**

Quantitative real-time PCR was used to investigate the effects of green tea (0, 1, and 2 g solid extract/kg diet) on the expression of *Ttp *family genes (*Ttp/Tis11/Zfp36, Zfp36l1/Tis11b, Zfp36l2/Tis11d, Zfp36l3*), pro-inflammatory genes (*Tnf, Csf2/Gm-csf, Ptgs2/Cox2*), and *Elavl1/Hua/Hur *and *Vegf *genes in liver and muscle of rats fed a high-fructose diet known to induce insulin resistance, oxidative stress, inflammation, and TNF-alpha levels.

**Results:**

Ttp and Zfp36l1 mRNAs were the major forms in both liver and skeletal muscle. Ttp, Zfp36l1, and Zfp36l2 mRNA levels were more abundant in the liver than those in the muscle. Csf2/Gm-csf and Zfp36l3 mRNAs were undetectable in both tissues. Tea (1 g solid extract/kg diet) increased Ttp mRNA levels by 50–140% but Tnf mRNA levels decreased by 30% in both tissues, and Ptgs2/Cox2 mRNA levels decreased by 40% in the muscle. Tea (2 g solid extract/kg diet) increased Elavl1/Hua/Hur mRNA levels by 40% in the liver but did not affect any of the other mRNA levels in liver or muscle.

**Conclusion:**

These results show that tea can modulate Ttp mRNA levels in animals and suggest that a post-transcriptional mechanism through TTP could partially account for tea's anti-inflammatory properties. The results also suggest that drinking adequate amounts of green tea may play a role in the prevention of inflammation-related diseases.

## Background

Recent investigations have established a mechanism for the regulation of inflammatory responses at the post-transcriptional level by tristetraprolin (TTP) family of CCCH tandem zinc finger proteins (ZFP). TTP family consists of three known members in mammals (ZFP36 or TTP, ZFP36L1 or TIS11B, and ZFP36L2 or TIS11D) and the fourth member in mouse and rat but not in humans (ZFP36L3) [1,2]. TTP (TIS11, G0S24, or NUP475), the best-studied family member, is the product of the immediate-early response gene *Zfp36 *in the mouse (*ZFP36 *in humans, refer to "Abbreviations" for the nomenclature of genes, mRNAs, and proteins) [3-5]. TTP binds to AU-rich elements (AREs) in some mRNAs and destabilizes those transcripts encoding proteins such as tumor necrosis factor-α (TNF-α) [6-9], granulocyte-macrophage colony-stimulating factor (GM-CSF) [10,11], and cyclooxygenase 2 (COX-2) [12]. The mRNAs encoding TNF-α and GM-CSF are stabilized in TTP knockout mice and in cells derived from them [8,10]. Excessive secretion of these cytokines in TTP knockout mice results in a severe systemic inflammatory response including arthritis, autoimmunity, and myeloid hyperplasia [13,14]. In contrast, up-regulation of TTP reduces inflammatory responses in macrophages [15]. These lines of evidence support the conclusion that TTP is an anti-inflammatory protein or arthritis suppressor.

Agents that induce *Ttp *gene expression may have potential therapeutic value for the prevention and/or treatment of inflammation-related diseases. A number of agents have been shown to increase Ttp mRNA and/or protein levels in mammalian cells. The inducible agents include growth factors (insulin, insulin-like growth factor I, epidermal growth factor, fibroblast growth factor, platelet-derived growth factor, and fetal calf serum) [3,4], cytokines (TNF-α, GM-CSF, and interferon-gamma) [4,5,8,15] and zinc [16]. However, *Ttp *gene expression is also induced by tumor promoters (phorbol 12-myristate 13-acetate and tetradecanoyl phorbol acetate) [3,5] and bacterial endotoxin lipopolysaccharide (LPS) [8,17,18]. Finally, *Ttp *gene expression is induced by viral infection [19]. The fact that most of these agents also increase the expression levels of pro-inflammatory cytokines such as TNF-α in the same cells and/or tissues [8,20] may limit the therapeutic potential of these agents. Therefore, it is important to search for other agents with the potential to up-regulate anti-inflammatory but down-regulate pro-inflammatory gene expression.

Tea (*Camellia sinensis*) is a popular beverage worldwide. Recent studies indicate that tea has a wide range of effects on animal and human health. A number of studies have indicated that green tea has anti-inflammatory properties [21]. Tea has been reported to have beneficial effects in conditions such as collagen-induced arthritis [22], inflammatory bowel disease [23] and carrageenan-induced paw edema [24]. However, the molecular mechanism of tea's anti-inflammatory property has not been completely elucidated. We hypothesized that TTP and/or its homologues might be involved in the beneficial effects of tea as an anti-inflammatory product.

A high fructose diet has been used as a model for the study of insulin resistance and oxidative stress in rats [25]. Overproduction of free radicals under oxidative stress is associated with inflammation. TNF-α levels are increased in rat muscle by high fructose diet, which is one of the determinants of insulin resistance in skeletal muscle [26,27]. A high fructose diet was also shown to activate inflammatory pathways in the liver [28]. Since liver and muscle are two of the most insulin-responsive organs and *Ttp *gene expression is induced by insulin [3], we hypothesized that TTP and/or its homologues might be involved in the inflammatory response induced by a high-fructose diet in rat liver and muscle, and that green tea might possess beneficial effects in high fructose-fed rats. Therefore, the demonstration of the effects of tea on inflammation-related gene expression in this model is important and complementary of its known antioxidant effects.

In this study, quantitative real-time PCR was used to investigate the expression profiles and the effects of green tea on the expression levels of the anti-inflammatory Ttp family mRNAs and some of the pro-inflammatory mRNAs known to be regulated by TTP family proteins. Our results showed that liver had more abundant Ttp family mRNA levels than skeletal muscle, and that Ttp mRNA levels were increased significantly and Tnf mRNA levels were reduced in the liver and skeletal muscle of rats fed a high-fructose diet and given 1 g of green tea extract/kg in the diet.

## Methods

### Animals, fructose-rich diet, and green tea extract

Male Wistar rats (6 weeks old, 150 ± 10 g) from Charles River Laboratories (Les Oncins, France) were housed individually in thermoformed polystyrene cages in accordance with standards accredited by the French Ministries of Agriculture and Environment. The rats were kept under the conditions with a 12-h light:12-h dark schedule, a room temperature of 21 ± 1°C, and a relative humidity of 55%. The fructose-rich diet used in this study contained 60% (w/w) fructose, 20.7% casein, 5% corn oil, 8% alphacel, 1% mineral mix, 1% vitamin mix, and 0.3% casein (SAFE, 89290, Augis, France). The green tea solid extract used in this study contained 12.75% (w/w) epigallocatechin-3-gallate (EGCG), 9.21% epigallocatechin (EGC), 3.73% epicatechin gallate (ECG), 2.4% epicatechin (EC), 5.94% caffeine, and 0.195% L-theanine (Unilevel France).

### Experimental design

All rats were fed a standard Purina chow for one week before being randomly divided into three groups with 10 rats per group. The first group of rats was given a high-fructose diet that has been shown to induce insulin resistance and oxidative stress (diet control). The second group of rats was given the high-fructose diet plus 1 g of green tea solid extract/kg diet (1 g tea) and the third group of rats was given the high-fructose diet plus 2 g of green tea solid extract/kg diet (2 g tea). Animals were sacrificed after 6 weeks on the diet. Food intake for rats fed the diet control, 1 g tea, and 2 g tea was 20.7 ± 0.8, 20.5 ± 0.9, and 20.3 ± 1.5 g/d, respectively. The body weight for rats fed the diet control, 1 g tea, and 2 g tea for 6 weeks was 360 ± 7, 350 ± 5, and 353 ± 7 g, respectively. The trend of decreased body weight in treated rats was not significant after 6 weeks of diet. The mean body gain by week for rats fed the diet control, 1 g tea, and 2 g tea was about 35, 34, and 34.5 g, respectively. These data are in agreement with a previous report in which the same diet was used and a similar evolution in body weight was reported [29]. The liver and skeletal muscle were removed from the rats, frozen in liquid nitrogen, and stored at -80°C. All procedures were in accord with guidelines of the National Institutes of Health and were approved by the French Army Ethical Committee.

### RNA isolation

Rat liver and muscle were ground into powder under liquid nitrogen. About 200–400 mg of the tissue powder was homogenized in 4 ml of TRI_ZOL _reagent (Invitrogen, Carlsbad, CA, USA) in a 15-ml Falcon tube. RNA was isolated according to the manufacturer's instructions. The RNA pellet was suspended in 20 μl DEPC-treated water. RNA integrity and concentrations were determined using RNA 6000 Nano Assay Kit and the Bioanalyzer 2100 according to the manufacturer's instructions (Agilent Technologies, Palo Alto, CA, USA) with RNA 6000 Ladder as the standards (Ambion, Inc., Austin, TX, USA).

### cDNA synthesis

Total cDNA synthesis was performed in 0.2-ml microfuge tubes using the ImProm-II Reverse Transcription System (Promega, Madison, WI). The reaction mixture (20 μl) contained 5 μg total RNA, 1 μg oligo(dT)_15 _primer (Invitrogen), 0.25 μg random primers (Invitrogen), 500 μM dNTPs, 5 mM MgCl_2_, 2.5 μl RNasin ribonuclease inhibitor, and 5 μl ImProm-II reverse transcriptase in 1× ImProm-II reaction buffer. The cDNA synthesis reactions were carried out at 42°C for 60 min.

### PCR primers and TaqMan probes

The primers and probes were designed using Primer Express software (Applied Biosystems, Foster City, CA, USA) and were synthesized by Biosearch Technologies, Inc. (Navato, CA, USA). The mRNA names, GenBank accession numbers, amplicon sizes, and the sequences (5' to 3') of the forward primers, TaqMan probes (TET – BHQ1), and reverse primers, respectively, are described in Table 1.

**Table 1:** Nucleotide sequences of real-time PCR primers and TaqMan probes

**mRNA**	**Accession No**	**Amplicon size**	**Forward primer (5' to 3')**	**TaqMan probe (TET – BHQ1)**	**Reverse primer (5' to 3')**
**Ttp/Tis11/Zfp36**	NM_011756	70 bp	GGTACCCCAGGCTGGCTTT	AACTCAATATAATCCTGCCTTAGCCTT	ACCTGTAACCCCAGAACTTGGA
**Zfp36l1/Tis11b**	NM_007564	60 bp	TGCGAACGCCCACGAT	ACCACCACCCTCGTGTCCGCC	CTTCGCTCAAGTCAAAAATGG
**Zfp36l2/Tis11d**	NM_001001806	77 bp	GAGGGCACCTCCCAACCT	TTGCAATTTCGACCATTACAGGACCCA	TGACAGAAGTGTGGTCGACATTT
**Zfp36l3**	NM_001009549	70 bp	CGAACTGCGTACCCTGTCAAG	CACCCCAAGTACAAGACGGAGCCTTG	GCCAACGCTGTGGAAGGT
**Tnf**	NM_013693	66 bp	GCTGTCGCTACATCACTGAACCT	TGCTCCCCACGGGAGCCG	TGACCCGTAGGGCAATTACA
**Ptgs2/Cox2**	NM_011198	106 bp	CCACCTCTGCGATGCTCTTC	AGCTGTGCTGCTCTGCGCT	CATTCCCCACGGTTTTGACATG
**Csf2/Gm-csf**	NM_009969	71 bp	CACCCGCTCGCCCAACCC	TCACCCGGCCTTGGAAGCATGTAGA	GGAGGCTCAGAGCTTCTTTGA
**Elavl1/Hua/Hur**	NM_010485	69 bp	CCTCCGAGCCCATCACAGT	AAGTTTGCAGCCAATCCCAACCAGAA	GCGAGAGGAGAGCCATGTTT
**Vegfa**	NM_001025250	68 bp	CAAAAACGAAAGCGCAAGAAA	CCCGGTTTAAATCCTGGAGCG	CGCTCTGAACAAGGCTCACA
**Vegfb**	NM_011697	83 bp	GATCCAGTACCCGAGCAGTCA	TGTCCCTGGAAGAACACAGCCAATGTG	TCTCCTTTTTTTTTGGTCTGCAT
**Rpl32**	NM_172086	66 bp	AACCGAAAAGCCATTGTAGAAA	AGCAGCACAGCTGGCCATCAGAGTC	CCTGGCGTTGGGATTGG

### Quantitative real-time PCR assays

The TaqMan reaction mixture (25 μl) contained 25 ng of total RNA-derived cDNAs, 200 nM each of the forward primer, reverse primer, and TaqMan probe, and 12.5 μl of 2× Absolute QPCR Mix (ABgene House, Epson, Surrey, UK). The reactions were performed in 96-well plates in a ABI Prism 7700 real-time PCR instrument (Applied Biosystems) [30]. The thermal cycle conditions were as follows: 2 min at 50°C and 10–15 min at 95°C, followed by 40–60 cycles at 95°C for 15 s and 60°C for 60 s. Fluorescence signals measured during amplification were considered positive if the fluorescence intensity was more than 20-fold greater than the standard deviation of the baseline fluorescence [30]. The ΔΔ*C*_*T *_method of relative quantification was used to determine the fold change in expression [31]. This was done by first normalizing the resulting threshold cycle (*C*_*T*_) values of the target mRNAs to the *C*_*T *_values of the internal control Rpl32 in the same samples (Δ*C*_*T *_= *C*_*T*Target _- *C*_*T*Rpl32_). It was further normalized with the diet control (samples with only the high-fructose diet but without tea supplement) (ΔΔ*C*_*T *_= Δ*C*_*T*Tea _- Δ*C*_*T*Diet_). The fold change in expression was then obtained (2−ΔΔCT
 MathType@MTEF@5@5@+=feaafiart1ev1aaatCvAUfKttLearuWrP9MDH5MBPbIqV92AaeXatLxBI9gBaebbnrfifHhDYfgasaacH8akY=wiFfYdH8Gipec8Eeeu0xXdbba9frFj0=OqFfea0dXdd9vqai=hGuQ8kuc9pgc9s8qqaq=dirpe0xb9q8qiLsFr0=vr0=vr0dc8meaabaqaciaacaGaaeqabaqabeGadaaakeaacqaIYaGmdaahaaWcbeqaaiabgkHiTiabfs5aejabfs5aejabdoeadnaaBaaameaacqWGubavaeqaaaaaaaa@33F1@).

### Statistical analyses

The data were analyzed by SigmaStat 3.1 software (Systat Software, Inc., Point Richmond, CA) using One Way Analysis of Variance (ANOVA). Multiple comparisons were performed with Duncan's Multiple Range Test. Values with different lower case and upper case letters displayed above the columns of the figures are significantly different at *p *< 0.05 and *p *< 0.01, respectively.

## Results

### Ttp family and other selected mRNA levels in rat liver and muscle

There are four forms of the anti-inflammatory TTP family proteins in rats and mice (TTP/ZFP36, TIS11B/ZFP36L1, TIS11D/ZFP36L2, and ZFP36L3) [1,2]. We examined the expression levels of these four forms of genes in the liver and skeletal muscle of rats fed a high-fructose diet known to induce insulin resistance and oxidative stress [25]. The *C*_*T *_values of the Ttp family and the internal control Rpl32 mRNAs and the relative ratio of Ttp family mRNAs in the liver and muscle are shown in Table 2. The *C*_*T *_values of Rpl32 in the liver and muscle were essentially the same and the results validated the assumption of using the Rpl32 mRNA level as an internal control for the normalization of gene expression levels. Ttp and Tis11b mRNAs were the two dominant forms of Ttp family messages in both liver and muscle. In liver, the relative levels of Ttp, Tis11b, and Tis11d mRNAs were 100%, 148%, and 6%, respectively. In muscle, the relative levels of Ttp, Tis11b, and Tis11d mRNAs were 100%, 70%, and 15%, respectively. Zfp36l3 mRNA was undetectable by 50 cycles of PCR in the liver or muscle. As a positive control for the PCR assay, Zfp36l3 mRNA was detected in two cell lines (data not shown). Ttp, Tis11b, and Tis11d mRNA levels were more abundant in the liver than those in the muscle, and were 5, 12.5, and 2 fold of those in the muscle, respectively (Table 2).

**Table 2:** Ttp family and other selected mRNA levels in rat liver and muscle

**Tissue**	**mRNA**	**Cycle of threshold (C_T _± SD)**	**Expression ratio (relative to Ttp) (Fold)**	**Expression ratio (relative to liver) (Fold)**
**Liver**	**Rpl32**	18.35 ± 0.40	Internal control	1.00
	**Ttp/Tis11/Zfp36**	21.23 ± 0.43	1.00	1.00
	**Zfp36l1/Tis11b**	20.67 ± 0.54	1.48	1.00
	**Zfp36l2/Tis11d**	25.19 ± 0.36	0.06	1.00
	**Zfp36l3**	0.00	0.00	
	**Tnf**	39.12 ± 0.77		1.00
	**Csf2/Gm-csf**	0.00		
	**Ptgs2/Cox2**	33.09 ± 0.39		1.00
	**Vegfa**	20.36 ± 0.27		1.00
	**Vegfb**	25.69 ± 0.56		1.00
	**Elavl1/Hua/Hur**	22.46 ± 0.22		1.00

**Muscle**	**Rpl32**	18.26 ± 0.26	Internal control	1.03
	**Ttp/Tis11/Zfp36**	23.81 ± 0.97	1.00	0.21
	**Zfp36l1/Tis11b**	24.32 ± 0.85	0.70	0.07
	**Zfp36l2/Tis11d**	26.51 ± 0.85	0.15	0.49
	**Zfp36l3**	0.00	0.00	
	**Tnf**	40.05 ± 0.55		0.59
	**Csf2/Gm-csf**	0.00		
	**Ptgs2/Cox2**	31.55 ± 0.83		2.42
	**Vegfa**	21.14 ± 0.35		0.59
	**Vegfb**	23.66 ± 0.29		4.27
	**Elavl1/Hua/Hur**	23.54 ± 0.18		0.42

Twenty-five ng of RNA-derived cDNAs were used for the quantitation of mRNA levels using 40–60 cycles of real-time PCR program. The mean C_T _(cycle of threshold) values and standard deviations (n = 5–8) are shown in the table. The relative ratios of mRNA levels were calculated using the double delta C_T _method normalized with Rpl32 C_T _value as the internal control and Ttp C_T _value or liver C_T _value as the calibrator.

Low levels of Tnf and Ptgs2/Cox2 mRNAs were detected in both liver and muscle (Table 2). Between these two tissues, Tnf mRNA levels in the liver were about 2-fold of those in the muscle. Conversely, Ptgs2 mRNA levels in the liver were about 40% of those in the muscle. The expression levels of Hu antigen R (Elavl1/Hua/Hur), vascular endothelial growth factor a (Vegfa) and Vegfb in the liver were about 2.5-fold, 2-fold, and 25% of those in the muscle, respectively. Csf2/Gm-csf mRNA was undetectable by 50 cycles of PCR in the liver or muscle. As a positive control for the assay, Csf2 mRNA was detected in mouse RAW264.7 cells (data not shown).

### Green tea increases Ttp mRNA levels in rat liver

**Figure 1:**
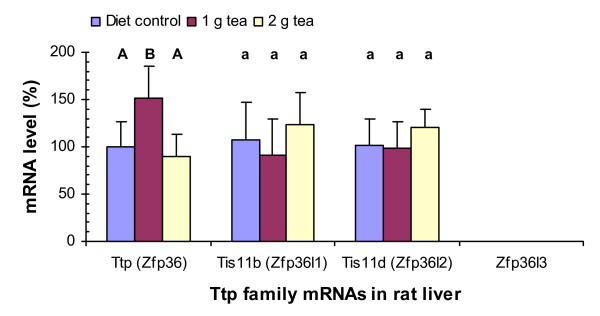
Green tea effects on Ttp family mRNA levels in rat liver. Total RNAs were isolated from livers of rats with metabolic syndrome induced by a high-fructose diet and reversely transcribed into cDNAs. Twenty-five nanograms of RNA-derived cDNAs were used for quantitative real-time PCR assays. The ΔΔ*C*_*T *_method of relative quantification was used to determine the fold change in expression. The results represent the percentage means and the standard deviations from 5–8 samples with 2–4 repetitions of each sample. Values with different upper case and lower case letters displayed above the columns of the figure are significantly different at *p *< 0.01 and *p *< 0.05, respectively.

Green tea (1 g solid extract/kg diet) increased Ttp mRNA levels by 50% (P = 0.003) but did not have statistically significant effects on Tis11b or Tis11d mRNA levels in the liver (Fig. 1). Green tea (2 g solid extract/kg diet) did not have statistically significant effects on Ttp, Tis11b, or Tis11d mRNA levels in the liver. Zfp36l3 mRNA was not detected by the PCR assays in the liver of rats treated with or without the tea supplement.

### Green tea increases Ttp mRNA levels in rat muscle

**Figure 2:**
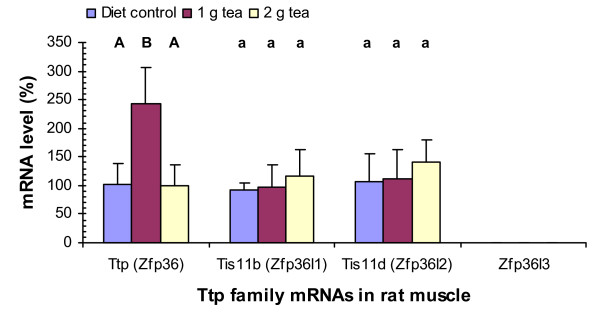
Green tea effects on Ttp family mRNA levels in rat muscle. RNA isolation, cDNA synthesis, real-time PCR assays, and statistical analyses were described in Fig. 1 legend.

Green tea (1 g solid extract/kg diet) increased Ttp mRNA levels by 140% (P < 0.001) but did not have statistically significant effects on Tis11b or Tis11d mRNA levels in the skeletal muscle (Fig. 2). Green tea (2 g solid extract/kg diet) did not have statistically significant effects on Ttp, Tis11b, or Tis11d mRNA levels in the muscle. Zfp36l3 mRNA was not detected by the PCR assays in the muscle of rats treated with or without the tea supplement. The percentage increases of Ttp mRNA in the muscle (Fig. 2) were higher than those in the liver (Fig. 1) but the net increases of Ttp mRNA levels in the liver were more than those in the muscle since Ttp mRNA levels in the liver were about 5-fold higher than those in the muscle (Table 2).

### Green tea decreases Tnf mRNA levels in rat liver

**Figure 3:**
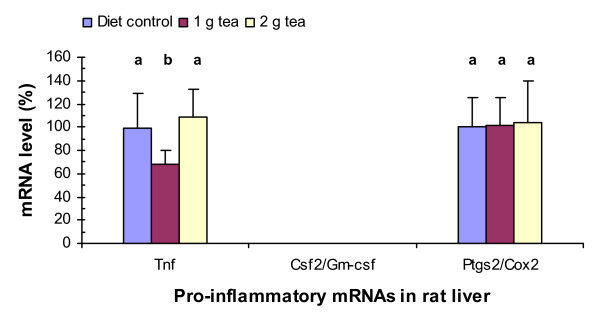
Green tea effects on Tnf, Csf2/Gm-csf, and Ptgs2/Cox2 mRNA levels in rat liver. RNA isolation, cDNA synthesis, real-time PCR assays, and statistical analyses were described in Fig. 1 legend.

The increased levels of the anti-inflammatory Ttp mRNA level with 1 g of green tea solid extract per kg of diet shown above suggest that green tea might have destabilizing effects on pro-inflammatory ARE-containing mRNAs such as Tnf mRNA. Therefore, we analyzed the expression levels of Tnf, Csf2/Gm-csf, and Ptgs2/Cox2 mRNAs, whose stability are known to be decreased by TTP [8,10,12], in the liver of rats fed with the high-fructose diet with or without 1 or 2 g of green tea solid extract per kg of diet (Fig. 3). PCR assays showed that green tea extract at 1 g decreased Tnf mRNA levels by 30% (P = 0.017) but did not have statistically significant effects on Ptgs2 mRNA levels in the liver. However, green tea extract at 2 g did not have statistically significant effects on Tnf or Ptgs2 mRNA levels in the liver. Csf2 mRNA was not detected by the PCR assays in the liver of rats treated with or without the tea supplement.

### Green tea decreases Tnf and Ptgs2/Cox2 mRNA levels in rat muscle

**Figure 4:**
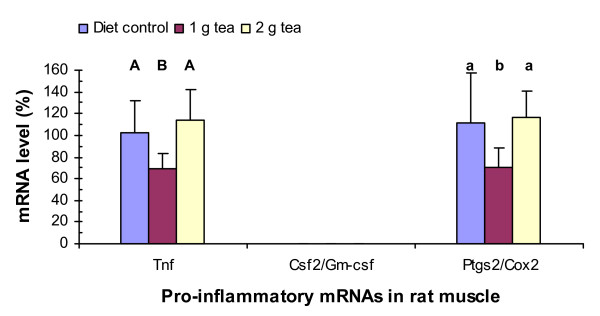
Green tea effects on Tnf, Csf2/Gm-csf, and Ptgs2/Cox2 mRNA levels in rat muscle. RNA isolation, cDNA synthesis, real-time PCR assays, and statistical analyses were described in Fig. 1 legend.

PCR analyses showed that green tea extract at 1 g decreased Tnf and Ptgs2 mRNA levels in the skeletal muscle by 30% (P = 0.008) and 40% (P = 0.042), respectively (Fig. 4). However, green tea extract at 2 g did not have statistically significant effects on Tnf or Ptgs2 mRNA levels in the muscle. Csf2 mRNA was not detected by the PCR assays in the muscle of rats treated with or without the tea supplement.

### Green tea does not affect Vegf mRNA levels in rat liver or muscle

Similar to Tnf and Ptgs2 mRNAs, Vegfa and Vegfb mRNAs are also ARE-containing mRNAs which are destabilized by TIS11B protein [32]. We investigated if green tea affected their mRNA levels in the liver and skeletal muscle of rats fed the high-fructose diet. PCR assays showed that green tea extract at 1 or 2 g did not have statistically significant effects on Vegfa or Vegfb mRNA levels in the liver or muscle (data not shown).

### Green tea increases Elavl1/Hua/Hur mRNA levels in rat liver

**Figure 5:**
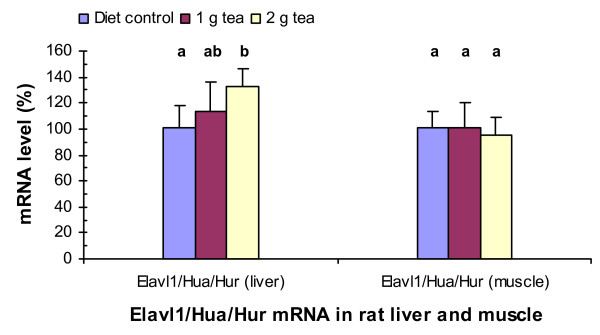
Green tea effects on Elavl1/Hua/Hur mRNA levels in rat liver and muscle. RNA isolation, cDNA synthesis, real-time PCR assays, and statistical analyses were described in Fig. 1 legend.

In contrast to TTP, ELAVL1 is known to be a stabilizing protein for some ARE-containing mRNAs [33-35]. We investigated if green tea affected Elavl1 mRNA levels in the liver and muscle of rats fed with the same high-fructose diet. Green tea extract at 1 g did not statistically significantly affect Elavl1 mRNA levels in the liver or muscle. However, green tea extract at 2 g significantly increased the mRNA levels by 40% (P = 0.007) in the liver but not in the muscle (Fig. 5).

## Discussion

TTP is an anti-inflammatory protein with the potential as a therapeutic target for the prevention and/or treatment of inflammation-related diseases. Previous studies have shown that Ttp mRNA and/or TTP protein levels are increased in mammalian cells by a wide range of agents. However, the fact that most of the same agents also increase the expression levels of pro-inflammatory cytokines in the same cells and/or tissues [8,20] limits these agents' therapeutic potential. In this study, we determined that green tea possessed the ability to increase *Ttp *but decrease *Tnf *gene expression in rats fed a high fructose diet known to induce insulin resistance, oxidative stress, and inflammation.

Tea is the most consumed beverage in the world and there are numerous reports of the health benefits of tea. One of the effects of tea is its anti-inflammatory property. In this study, we explored the relationship between green tea and the mRNA levels of some pro-inflammatory and anti-inflammatory genes in the liver and muscle of rats with metabolic syndrome induced by a high-fructose diet. We demonstrated that a low dose of green tea (1 g solid extract/kg diet) significantly increased the mRNA levels of the anti-inflammatory protein TTP and decreased those of the pro-inflammatory TNF-α in both liver and muscle of rats and also decreased Ptgs2 mRNA in the muscle. The induction of Ttp and reduction of Tnf mRNAs by green tea in rats suggests that at least part of the mechanisms of tea's anti-inflammatory effects may be involved in TTP at the post-transcriptional level.

Previous investigations have suggested that the mechanism of tea's anti-inflammatory effects also involves the regulation of gene transcription. Tea affects a number of important molecular targets including TNF-α [36], interleukin 2 (IL-2) [23], signal transducer and activator of transcription-1α (STAT-1α) [37], inducible nitric-oxide synthase (iNOS) [38], nuclear factor-kappa B (NF-κB), insulin activity [39-41], and affects DNA and RNA directly [42]. TNF-α plays a crucial role in inflammation. NF-κB, an oxidative stress-sensitive nuclear transcription factor, controls the expression of *Tnf *and other genes. It was shown that green tea polyphenol EGCG inhibited LPS-mediated Tnf mRNA levels by blocking NF-κB activation in macrophage RAW264.7 cells [36]. Therefore, it was proposed that the anti-inflammatory mechanism of green tea polyphenols is mediated at least in part through down-regulation of *Tnf *gene expression by blocking NF-κB activation [36]. Taken together, the molecular mechanism of tea's anti-inflammatory effects may be involved in the down-regulation of pro-inflammatory cytokines such as TNF-α at both transcriptional and post-transcriptional levels [8,17,18].

We also found that a higher dose of green tea extract at 2 g in the high-fructose diet significantly increased the mRNA levels of Elavl1/Hua/Hur in rat liver. ELAVL1 is known to be an RNA binding protein [33-35]. Previous studies suggest that ELAVL1 stabilizes ARE-containing mRNAs [34], but a recent study suggests that ELAVL1 in murine innate compartments suppresses inflammatory responses in vivo by inducing the translational silencing of specific cytokine mRNAs [33]. This study suggests that a higher dose of green tea might also have beneficial effects on reducing inflammation via ELAVL1 in these tissues. Vegfa and Vegfb mRNAs are known to be destabilized by TIS11B protein [32]. Our study showed that Tis11b and Vegf mRNA levels in rat liver or muscle were not affected by either tea dose. These results suggest that Vegf mRNAs may be the physiological targets of TIS11B rather than TTP in these tissues.

There are two known TTP-related proteins in mammalian cells, ZFP36L1 (TIS11B, cMG1, ERF1, BRF1, or Berg36) and ZFP36L2 (TIS11D, ERF2, or BRF2) [1]. A third related protein (ZFP36L3) was recently identified that seems to be expressed only in rodents as a placenta-specific protein [2], and the expression in other species has not been reported. These four proteins are all capable of binding to and destabilizing ARE-containing mRNAs in vitro and in transfected cells [1,1,43]. However, gene knockout studies have provided evidence for their unique roles in vivo. TTP knockout mice develop a severe inflammatory syndrome [13,14]. Mice deficient in ZFP36L1 develop chorioallantoic fusion defects and embryonic lethality [44]. Mice with decreased levels of an amino-terminal truncated form of ZFP36L2 exhibit female infertility and disrupted early embryonic development [45]. Despite their unique contributions, information is limited on the expression profiles of Ttp family mRNAs in the same tissue and/or in different tissues.

We therefore described the expression profiles of the four Ttp family mRNAs in rat liver and muscle. Real-time PCR analyses showed that Ttp and Tis11b mRNAs were the two major forms in the liver and muscle and that Ttp, Tis11b, and Tis11d mRNA levels were more abundant in liver than those in muscle. The PCR results are in agreement with immunoblotting results reported previously that TTP protein levels are more abundant in mouse liver than muscle [17]. Zfp36l3 mRNA levels in the liver or muscle were below the detection limit even after 50 cycles of PCR amplification. These PCR results are in agreement with those of the Northern blot results reported previously to be specific to placenta and extra-embryonic tissues in mouse [2]. However, these expression profiles of Ttp family mRNAs in the rat liver and muscle are somewhat different from those in cultured human monocytes, in which the mRNA levels of these three forms are similar in un-induced monocytes [46].

The induction of Ttp and reduction of Tnf mRNAs in rat liver and muscle by green tea (1 g solid extract/kg high fructose diet) suggests that TTP may be involved at the post-transcriptional level in the mechanisms of tea's anti-inflammatory effects. However, some issues need to be investigated in future experiments. First, the reason(s) why tea at 2 g solid extract/kg diet did not increase Ttp mRNA levels and decreased Tnf mRNA levels in the same tissues requires further studies to determine the optimum dose that demonstrates green tea effects. It is possible that *Ttp *gene expression is regulated by a narrow dose of tea for certain period, since its gene expression is transiently induced by growth factors such as insulin, i.e., Ttp mRNA levels peaked at 45 min but declined to normal level in 2 h in a cell culture system [3]. Furthermore, the effect of green tea on insulin levels in the plasma showed a similar pattern, i.e., 2 g tea treatment was less effective than 1 g tea treatment. The insulin levels were 412 ± 100 pmol/l, 113 ± 28, and 197 ± 43 for the diet control, 1 g tea, and 2 g tea treatments for 6 weeks [47]. Therefore, it is important to conduct more extensive analysis of the dosage effect and time course of tea's effect on *Ttp *and *Tnf *gene expression. We believe cell culture systems could be a much more effective way to address this area of research in the future. Second, confirmation of TTP protein levels in rats with tea treatments requires future development of high-titer rat antibodies, since TTP protein is only detectable with the mouse TTP antibodies in the spleen of rats after LPS stimulation [8,17,18] or in rats following dexamethasone induction [48]. Third, it's of interest to determine if the change of Tnf mRNA level correlates with the change of TNF-α protein level. However, since TNF-α is a secreted protein, a cell culture system may be more suitable to do detailed analyses of the TNF-α protein levels and would allow a detailed time course of the TNF-α protein levels. Fourth, it will be interesting to investigate if green tea has any beneficial effects in rats fed a normal diet. However, we speculate that there might be large differences in gene expression in rats treated with a high-fructose diet and a normal diet (no fructose), since the dietary environment between rats fed a normal diet and those fed a high fructose diet is dramatically different. Finally, what is the mechanism(s) of green tea activation of *Ttp *gene expression? Our working hypothesis is that *Ttp *gene expression is increased by catechins, the major polyphenols in green tea. We reported that polyphenolic compounds in cinnamon increased TTP protein levels in mouse 3T3-L1 adipocytes [49,50]. Recently, it was demonstrated that tea catechins directly bind to DNA and preferentially bind to poly(A), poly(U), and poly(AU) in RNA [42]. It will be interesting to investigate if green tea polyphenols could bind to the regulatory elements of *Ttp *gene and/or bind to the AU-rich elements in the 3'-untranslated region of Tnf mRNA, which are also the preferred binding sites for TTP protein [51,52].

## Conclusion

This study describes profiles of the anti-inflammatory Ttp family mRNA levels and green tea effects on these and some of the pro-inflammatory mRNAs in the liver and muscle of rats with metabolic syndrome induced by a high-fructose diet. Our results suggest that the molecular mechanism of the anti-inflammatory effects of green tea may be partially due to its ability to increase mRNA levels encoding anti-inflammatory factors such as TTP/TIS11/ZFP36 and/or ELAVL1/HuA/HuR and to decrease mRNA levels encoding pro-inflammatory factors such as TNF-α and/or PTGS2/COX-2. To our knowledge, this is the first report to show that a plant nutritional product such as green tea can modulate Ttp mRNA levels in a biological system and the results provide a novel post-transcriptional mechanism for green tea's anti-inflammatory properties. These results suggest that drinking adequate amounts of green tea may play a role in the prevention of inflammation-related diseases.

## Abbreviations

ANOVA: one way analysis of variance; ARE: AU-rich element; COX-2/PTGS2: cyclooxgenase-2/prostaglandin-endoperoxide synthase 2; EGCG: Epigallocatechin-3-gallate; GM-CSF/CSF2: granulocyte-macrophage colony-stimulating factor; HuR(HuA)/ELAVL1: Hu antigen R/embryonic lethal, abnormal vision-like 1; LPS: lipopolysaccharide; NF-κB: nuclear factor-kappa B; RPL32: ribosomal protein L32; TNF: tumor necrosis factor; TTP: tristetraprolin; VEGF: vascular endothelial growth factor; ZFP36: zinc finger protein 36; ZFP36L1: ZFP36-like 1; ZFP36L2: ZFP36-like 2; ZFP36L3: ZFP36-like 3. The nomenclature of genes, mRNAs, and proteins was according to reference 1 and was accepted by the Mouse Genome Database. For example, *Zfp36*, Zfp36, and ZFP36 represent Zfp36 gene, mRNA, and protein, respectively.

## Competing interests

The author(s) declare that they have no competing interests.

## Authors' contributions

HC designed the experiment, designed PCR primers and probes, performed PCR assays, analyzed PCR data and wrote the manuscript. MAK performed RNA isolation and cDNA synthesis. FK and HDD provided advice on RNA isolation, cDNA synthesis, and PCR assay. JFU analyzed the data and revised the manuscript. SC provided the green tea solid extract. AMR designed the animal study and provided the animal tissues. RAA designed the animal study, analyzed the data, and revised the manuscript.
